# Practical Observations on Fever, Dysentery, and Liver Complaints, as They Occur Amongst the European Troops in India; with Introductory Remarks on the Disadvantages of Selecting Boys for India Military Service. Illustrated by Numerous Cases and Tables

**Published:** 1819-07-01

**Authors:** 


*Practical Observations on Fever, Dysentery, and Liver Complaints, as they occur amongst the European Troops in India; with Introductory Remarks on the Disadvantages of selecting Boys for India Military Service. Illustrated by numerous Cases and Tables.* By GEORGE BALLINGAL, Esq. Surgeon to His Majesty's 33d Regiment of Foot. 1 Vol. 8vo. pp. 248. Edinburgh, 1818.

TILL within these few years, the Medical Topography and the Diseases of our vast Asiatic possessions, were comparatively unknown to the European part of the profession. Our medical brethren in those sultry regions, though many of them had both the leisure and the means of prosecuting pathological and therapeutical studies, contented themselves in general, with a faithful discharge of their own immediate duties, while a wide separation from one another, and from the mother country, gradually extinguished that ardent zeal which is so necessary for investigations pointing ultimately to the general advancement of medical science. The late war, however, which called forth the collected and individual energies of nations, produced a corresponding effect on the medical corps attached to our fleets and armies in the different quarters of the globe. Hence, there is scarcely a subject in medicine or surgery, which has not been elucidated or improved, by the class of medical society abovementioned, as the works that are daily issuing from the press abundantly prove.

The book under review is one of these. It is written with great modesty, and apparently *presents* a faithful record of our author's individual experience, without any allusion to, or assistance from, the works of others. We shall, therefore, give a full analysis of its contents.

In the introductory paper Mr. B. reprobates the plan of sending boys to India before their habits are formed, and their frames arrived at maturity. Every one knows how unfavourable a tropical climate is for training young soldiers to habits of activity and military discipline; while, with daily opportunities of witnessing the irregularities and frequent intoxication prevalent amongst the older soldiers in that country, it cannot be expected they will long refrain from a participation in their excesses. Hence, our author thinks, it is surely inconsistent to remove men at this early period of life from their native climate, which admits of abundance of salutary exertion at all hours of the day, to a country where the period of exercise is necessarily limited to a few hours in the morning and evening, and where the remainder of the day is too generally devoted by the soldiers to drinking and dissipation. But we shall leave this subject for those whom it may concern, and come to the purely medical part of the work.

### FEVER.

This is not a prominent feature in the returns of an Indian army; yet when we contemplate the extent of visceral obstructions among all ranks, most of whom trace their complaints to previous attacks of fever, we cannot but regard its *sequelæ* as infinitely more formidable than itself. From observation and inquiry, Mr. B. has been led to believe that the treatment which has proved sufficient to prolong a patient's existence, is by no means competent to the restoration of his health. Our author's object, therefore, in this paper, is to press the necessity of a vigorous use of evacuations at the commencement of the disease, "and to urge the liberal use of purgatives, blood-letting, and the cold affusion."

In a period of nearly seven years, the regiment to which our author belonged lost but forty-two men from fever; and of eighty-seven cases, the notes of which were preserved, eighty-six terminated favourably. Here too, the fatal case exhibited abscess in the liver. During the months of September and October, 1810, fever prevailed, to a very great extent, in a detachment of which he had the medical charge at Masulipatam; "and by the liberal use of purgatives, cold affusion, and blood-letting, he succeeded in conducting every one of the cases to a favourable termination, and in preventing the occurrence of any of the dreadful sequelæ of this disease." Our author acknowledges candidly, that these fevers are not to be compared with the more formidable fevers of Batavia, Bengal, Bencoolen, &c. yet he is convinced that the difference is more in degree than in kind; and he is led to infer that a more extended use of the practice which has been so eminently successful in these milder forms of fever, would be equally beneficial in the more destructive fevers of other parts of India.

The fever which our author witnessed on the Madras establishment was that characterized by many authors under the name of bilious remittent. Its approach was marked by a peculiar degree of listlessness and inaction; a sense of weight and pain in the forehead; pains in the loins and lower extremities; extreme lassitude and prostration of strength;—in plethoric young men, a flushing of the face, with fullness and suffusion of the eye balls. The heat of surface often becomes intense, particularly about the præcordia, with a greater degree of febrile oppression, anxiety, and restlessness, than in fevers of a graver type. The tongue generally appears furred, and covered with a yellow crust; the taste is disagreeable; the fauces parched; the patient is distressed with insatiable thirst; loathing of food; retching, and vomiting of discoloured bile. The bowels are generally costive, while a sense of fullness, tension, and tenderness is often perceptible in the abdomen. The evacuations, when they appear, are generally dark coloured, and peculiarly fœtid; the urinary discharge is extremely high coloured, with strong sense of scalding in voiding it. Mr. B. observes that the appearance of vitiated and redundant secretion of bile, conspicuous in the alvine and urinary evacuations, the turgescence, tension and tenderness of the abdomen, the extreme restlessness, anxiety, and impatience of one position, all evince a disposition to congestion in the glandular viscera of the belly, in which consists the chief source of danger in this disease. In the few cases where our author had an opportunity of examining the appearances on dissection, "the brain, the liver, and spleen have seemed to be the organs particularly affected; an increased flow of blood, and apparent tumefaction in one or all of these organs has invariably been remarkable. In the brain this has been marked by an obvious distention of the superficial blood-vessels on its surface, while in the liver and spleen, where the vessels are embedded in the parenchymatous substance of these organs, it has been marked by a general increase of size and weight, and the effusion of an unusual quantity of blood on cutting into their substance." 25.

Mr. B. justly states here, that these phenomena are common where people are cut off in the early stages of all fevers; "but what I wish, says he, particularly to remark, is a disposition to congestion in the liver, which the nature of the climate, and the dissipated lives of the soldiers in India, perhaps tend to fix particularly on this viscus; and in many cases, when the fever does not immediately prove fatal, it nevertheless terminates in a permanent disease of that organ, laying the foundation of a chronic flux; and after a lapse of weeks and months, ultimately carrying off the patient, by the supervention of abscess in the liver, or of inflammation and mortification in the course of the large intestines." Of forty-two cases of fever, noticed in the register, several terminated in this circuitous manner, as did many of the Batavian and Seringapatam fevers. The number is also considerable in which the fevers under rewiew end in fluxes, and in abscesses of the liver. We now come to our author's therapeutics.

1. *Purgatives.* To many of the Indian diseases, this class of medicine seems peculiarly appropriate; but to none more than to fever. The constipation and vitiated stools render purgatives indeed indispensible. Mr. B. does not so highly prize mercurial purgatives, as the practitioners of India, in general, do; and, indeed, on some few points, we have thought that we could perceive a disposition in our author to deviate from general opinion, rather through desire of singularity than strength of argument adduced in favour of his tenets. Jalap and salts were the purgatives from which our author derived the greatest benefits in the commencement of fever. We need not say that irritability of stomach is a thing both to be dreaded and obviated, if possible; and that, on this account, the mercurial aloetic medicines are the best upon the whole. Injections are to aid the purgatives, and when all accumulations are removed, the bowels are to be kept lax by diluted solutions of the neutral salts, or mineral waters, until the febrile symptoms entirely disappear; until all sense of tenderness of the abdomen subsides, and a soft and natural feel of its parietes evinces the return of a healthy disposition in the organs within.

2. *Blood-letting.* "An extreme aversion to the use of the lancet is a trait remarkably conspicuous in the practice of the older Indian physicians." "I may, however, safely assert, that neither in fever nor in any other disease, have I ever had occasion to regret the employment of blood-letting; while, on the other hand, I have frequently had to blame myself for its omission." 30. It appears to our author, that although youth, plethora, vigour, and recency from Europe are circumstances which undoubtedly enable men to bear bleeding to a much larger extent than under opposite circumstances, "yet this evacuation becomes no less necessary in older men, to obviate the tendency of the disease to fix upon and impair the functions of the liver, from which an increase of years certainly affords them no exemption, and to which their residence in India, if of any duration, and the habits of the soldiery there, as certainly create a positive predisposition." Mr. B. thinks that *here*, though a copious detraction of blood be not necessary, yet that the loss of twenty or thirty ounces will frequently prove sufficient to prevent congestion or inflammation of internal organs, especially if combined with purgatives. To this extent our author has been in the habit of going in the oriental fevers; and to this measure he attributes, in a great degree, "the very general exemption of his patients from those visceral obstructions so often consequent on this disease." Where determinations of blood to the head were evident, temporal arteriotomy, or leeches to the head were practised, in addition to the venesection. When tenderness or pain in the region of the liver indicated an incipient affection of that organ, blisters were resorted to.

3. *Cold Affusion.* Our author lauds this measure highly in the fevers of India; but we fear its day is fast drawing to a close; we shall therefore pass the measure in silence.

4. *Mercury,* he thinks, unnecessary in the common fevers of the Madrass presidency, if the liberal evacuations already alluded to are used.

"Where, however, the patient has laboured under an indifferent state of health previous to the accession of urgent febrile symptoms, and where the use of purgatives alone seemed inadequate to the restoration of healthy secretions, and natural alvine evacuations, I have frequently had recourse to it with the very best effect; and in the more severe forms of fever prevalent in India, it is a remedy reckoned indispensible. *While some difference of opinion exists regarding the mode of its operation, there seems to be but one opinion generally prevalent regarding its extensive utility.*" 35.

In the sequelæ of these fevers, this medicine, of course, is also indispensible; especially where a variable and uncertain appetite, an irregular state of the bowels, a foul tongue, a pearly eye sometimes tinged with yellow, a sallow complexion, and a parched skin, give additional reason to suspect that the liver is in fault.

"And although no pain in the region of the liver should guide us to the seat of the disease, we are to proceed as if it were the organ principally affected."

Mercury, according to our Author, is here a remedy in which he places the most unbounded confidence; especially when assisted by change of climate, or a temporary absence from the station where the disease was contracted.

"Long continued, or even repeated courses of ptyalism, succeeded by a journey to the sea coast, or a trip to sea, will frequently succeed in restoring a patient to a competent share of health, and enabling him to resume his duties in India; and should these fail, we have then no resource left but to recommend a return to Europe."

5. *Emetics.* The gastric irritability so common in the fevers of this and other intertropical regions, deterred, and well it might, our author from emetics; he therefore used them very rarely, and very sparingly.

6. *Bark.* In the remittent fevers of India, our author has no estimation for cinchona; and even "in the treatment of intermittent fevers, (says he) for which it has been so extensively used, I have not found its exhibition attended with that decided success which many would lead us to expect. And as these fevers are so generally dependent upon, or at least connected with, organic disease of the abdominal viscera, we may fairly be allowed to doubt the efficacy of bark, or the propriety of its exhibition; and these doubts are fully confirmed by all the experience we had in the Royals, particularly at Trichinopoly, where this disease prevailed to a great extent; and where it was, in many instances, removed by the solution of arsenic, after the bark had failed." 39.

Thus concludes our author's paper on Fever; and we need not inform our Readers, that had Mr. Ballingal condescended to read a few modern books, he would have found all his observations completely anticipated. Every corroboration, however, is valuable; and as such, we have great pleasure in pourtraying the author's sentiments. We shall now proceed to the second division of the work.

### DYSENTERY.

Our author objects, in limine, and well he may, to Dr. Cullen's definition of dysentery, at least as it appears in India, and, we may add, in Europe. In our eastern colonies, he observes, this disease often makes considerable devastation on the intestinal canal before pyrexia becomes evident; and as to its contagious nature, it is totally unnecessary to mention the absurdity of the opinion, once so currently adopted, and even yet pertinaciously retained by a few individuals. "The appearance of scybalæ, another striking feature of the disease, as characterised in Europe, is comparatively a rare occurrence in India." We would ask Mr. Ballingal, if his own *post mortem* examinations, or personal observations, confirm this story of scybalæ in European dysentery? We can only say, that during the late war, chance threw us in the way of opening, and seeing opened, some hundred bodies who died of dysentery in Europe; and we can safely assert, that scybalæ are as infrequent here as in India. We thus see how error may be propagated. The above distinction, which exists only in books, and in Mr. Ballingal's imagination, is brought forward as a proof that tropical and Hyperborean dysentery are different diseases!

Dysentery then, in our Indian territories, is divided into two varieties; colonitis, (not necessarily implying the existence of any discharge from the bowels) and hepatic flux.

It is colonitis, which, according to our author, makes the greatest ravages, *at first*, among the European troops. The causes are conceived to be "Heat, particularly when combined with moisture; the immoderate and indiscriminate use of fruits; the abuse of spirituous liquors; exposure to currents of wind and noxious night dews." But without attempting to account satisfactorily for colonitis, our author proceeds to a description of the disease, taken from the Critical Review for 1802, as given in the extract of a letter to Sir Walter Farquhar. The writer of this letter states, that the disease is attended from the beginning with a severe fixed pain above the pubes, attended with extreme difficulty of making water, and frequently an entire suppression of urine. There is, at the same time, a violent and almost unceasing evacuation from the bowels, of a matter peculiar to the disease, and which exactly resembles the washings of raw flesh. High fever, unquenchable thirst, and perpetual watching, attend the complaint. The pulse is hard and strong, resembling that in the highest degree of pleurisy, or acute rheumatism. The fixed pain above the pubes; the peculiar evacuation; and the suppression of urine, may, it is thought, be considered pathognomonic of this disease. From dissection, the colon seems to be primarily affected; and the bladder suffers only from communication, as the lower part of the large intestine is generally inflamed. "Bleeding seems useful; but opium given in the commencement is the most effectual remedy." If omitted till the fever supervenes it is injurious, and can only be administered towards the decline of the disease. The remedies then are emollient glysters and drinks, with fomentations above the pubes, which are more useful than blisters.

It has only happened to Mr. Ballingal to meet the disease with these highly inflammatory symptoms, where it occurred as a consequence of hard drinking, and where it had made considerable progress before medical assistance was called in.

"The form of flux, now under consideration, commences in general with much of the appearance of a common diarrhœa; frequent and unseasonable calls to stool, with an irresistible inclination to strain over it. The evacuations are generally copious, of a fluid consistence, without any peculiar fætor; sometimes streaked with blood, and at other times a small quantity of blood is voided in a separate form, unmixed with the fæcal matter. The pulse, in this stage of the disease, is seldom altered; the heat of the skin not perceptibly increased, and the tongue is frequently but little changed in its appearance. There is always a great prostration of strength and depression of spirits; the former symptom being always strongly dwelt upon by the patient; the appetite is indifferent, and the thirst urgent. To these symptoms succeed a fixed pain in the hypogastrium, more or less acute; the pain sometimes extending to, and peculiarly urgent in one or both the iliac regions; and sometimes to be traced along the whole course of the colon, with a sense of fulness, tension, and tenderness upon pressure; and on applying the hand to the surface of the abdomen, a preternatural degree of heat is frequently perceptible in the integuments; the evacuations now become more frequent, and less copious; they consist chiefly of blood and mucus, or are composed of a peculiar bloody serum, which has very aptly been compared to water in which beef has been washed or macerated. A suppression of urine and distressing tenesmus now become urgent symptoms; the indifference to solid food increases, while there is an uncontrollable desire for liquids, particularly cold water, which the patient prefers to any drink that may be offered to him, and from which he expresses his inability to refrain, although prepossessed with the idea of its being injurious. The tongue is now generally white, and furred, sometimes, however, exhibiting a florid, smooth, and glassy appearance, with a tremulous motion when thrust out; the skin is either parching hot, so as to render it even painful to retain the hand in contact with it, or covered with profuse perspiration, insomuch that it may often be observed standing in large drops on the surface; the pulse is still frequently but little affected, sometimes, however, it assumes a febrile quickness, without any other remarkable feature; at other times it will be found without any increase of velocity, but full, and bounding with a peculiar thrilling sensation under the fingers. This state of the pulse, whenever it takes place, always denotes extreme danger, and shews that the disease is rapidly hurrying on to the final stage, in which the lassitude and dejection, so conspicuous throughout its course, are now converted into the utmost degree of anxiety, depression, and fear of death. The patient generally shews an inclination to dwell upon symptoms, which to a spectator would appear of minor importance. He evinces the greatest reluctance to part with his medical attendant, though fully sensible how unavailing the efforts of medicine are likely to prove, The discharges by stool, which are frequently involuntary, are now accompanied with the most intolerable fœtor; they are frequently mixed with shreds of membrane, and quantities of purulent matter; a protrusion of the gut, forming a complete procidentia ani often takes place; and cases are not wanting, where a portion of the inner coat of the intestines, amounting to some inches, has been thrown off in a state of mortification." P. 49.

When things come to this pass, death soon closes the scene. The periods occupied in passing through the different stages are very various, the disease often proving fatal within a week—and at other times being protracted to two or three weeks, but seldom longer, where the inflammation is solely confined to the colon.

Our author next proceeds to the symptomatology of the more chronic form of disease, denominated "Hepatic Flux." This is more incident to men after some residence in India, and particularly those who are prone to irregular and disordered secretions of bile. It often, like the other, assumes the form of diarrhœa at first, and becomes afterwards characterized by frequent and severe fits of griping, resembling cholic pains, particularly urgent about the umbilical region. The evacuations at the beginning always exhibit something unnatural in their colour, varying from the darkest inky hue to the different degrees of green and yellow; all these colours often alternating. The stools are accompanied with much flatus, and generally exhibit a frothy appearance, attended with a sense of scalding about the anus, the patient enjoying an interval of ease after each evacuation. The intervals, however, are generally so short, that the soldier often prefers carrying a mat with him to the necessary, in order to pass the night there, rather than have to run backwards and forwards to the barrack-room.

"From the commencement the patient complains of nausea, inappetency; preternatural thirst; bad taste in the mouth, the tongue being furred, and loaded with a bilious crust; the pulse quickened and the skin parched. After a few days, the stools become white, passed with straining, and mixed with half digested aliment. The complaint is now denominated by the soliers "the white flux," and its obstinacy is well known among them. The griping pains continue, and sometimes the patient feels a permanent degree of oppression about the epigastric region. Nausea, hiccup, and bilious vomiting now become highly distressing; the thirst becomes urgent, with lassitude, debility, and increasing emaciation. The skin often communicates a greasy sensation to the touch. In this way the patient goes on for weeks, or even months, the complaint terminating in recovery, or the patient is carried off by an abscess in the liver, or by the accession of ulceration and mortification in the course of the colon; the accession of the latter is to be apprehended, from the appearance of blood in the stools, and the other symptoms of colonitis formerly detailed." P. 53.

*Post-mortem Appearances in Colonitis.* Inflammation of that part of the tube situated below the valve of the colon. No disease of the structure of the liver.

"Yet I am by no means disposed to infer, from the want of morbid appearances in the liver, that this viscus may not have been, in many cases, the seat of *diseased action*, during the life of the patient." P. 53.

When the abdomen is laid open, an effusion of serum, sometimes mixed with coagulable lymph, is found accumulated in this cavity; the omentum generally shrunk, firmer than usual, and of a doughy feel, with slight adhesions to the convolutions of the intestines; at other times shrivelled and destitute of fat. The stomach seldom altered in its appearance. The small intestines sound, sometimes exhibiting slight inflammatory patches adhering to the omentum. No peculiar appearance on the inner coat of the small intestines.

"The great intestines again, the principal seats of disease, shew the strongest marks of inflammation in all its stages; some portions exhibiting externally a slight inflammatory redness, while others are marked by the highest degree of lividity; and in some cases, parts of the gut will be found to have given way, so as to permit the escape of air, and even of fæces, into the cavity of the abdomen; and in these destructive effects of inflammatory action, the cæcum, with its appendix vermiformis, and the sigmoid flexure of the colon, will, in most cases, be found to participate largely." P. 55.

The appearance of cells in the colon is, in a great measure, obliterated, and the coats of the intestine often so thickened throughout, as to suggest the idea of a solid rope; and so much altered in tenacity—so brittle in texture, as not to admit of being handled without risk of rupturing them. The calibre of the gut is found much diminished by the thickening of the coats; the villous coat in some places abraded simply, in others ulcerated, and besmeared with bloody mucus, mixed with specks of pus. In some places, this coat of the colon exhibited a tuberculous appearance, not inaptly compared to small-pox. Extravasated grumous blood is not unfrequently found in the colon. Scybalæ very seldom met with. The liver sometimes free from apparent disease, at other times preternaturally small and indurated, or enlarged and hardened; the bile unhealthy looking. The other viscera not often or materially affected, excepting the mesenteric glands, which are frequently found enlarged and obstructed.

Mr. Ballingal having endeavoured to establish the existence of two distinct modifications or varieties of India flux, proceeds to the treatment, which is very briefly detailed. He considers the colonitis, or acute form of flux, as a local disease, unconnected with the liver or the constitution—an inflammation, in short, of the large intestine, tending rapidly to mortification.

"But even allowing that a diseased action of the liver, and a vitiated state of the biliary secretion, have always preceded the attack of colonitis, and have been, in some measure, the cause of the latter affection, still, in the state we meet the disease, the effect appears to have greatly outrun the cause; they bear no adequate proportion to each other; and it is too much to expect that, by taking away the one, the other will cease." P. 66.

We do not conceive that this is very good reasoning. The disordered function of one organ will sometimes produce diseased structure in another, which is infinitely worse; yet the latter might have been prevented, [not indeed cured] by removing the former. We have no objection, indeed, to our author's mode of obviating the local affection of the intestine, whether it be primary or secondary; but if the *latter*, the original source of the mischief ought also to be sought after, and, if possible, removed.

Mr. B. then recommends the vigorous use of topical remedies, as leeches, blisters to the surface of the abdomen, fomentations, anodyne and astringent injections. But our author does not reject the use of general remedies, and particularly of *blood-letting*. Of this measure he entertains a very favourable opinion. He candidly owns, however, that this opinion "is grounded more on the ravages of inflammation, so universally apparent in the dead, than on any repeated or extensive experience of its beneficial effects on the living." In the regiment where our author served, there was a general dislike to the use of the lancet; as, indeed, there was, and we fear still is, among "the older practitioners in India." Moreover, a long voyage of full five months, without having touched any where for refreshments, had lowered the tone of the European constitution, so that by the time they had got over the first objection, from repeated experience of the safety and utility of bleeding in other diseases, the period for its employment in dysentery was past, or at least rendered extremely questionable, while the complicated nature of the cases latterly occurring lessened its necessity.

"In short, (says Mr. B.) of the few cases of dysentery in which I have employed bleeding, the majority have, I think, terminated favourably; and of those in which the result has been fatal, the appearances on dissection have been such as to excite a sentiment of regret at not having carried the evacuation farther." P. 68.

*Purgatives*, Mr. B. exhibits at the very beginning, in order to clear the alimentary canal, and ascertain the state of the fæcal matters. If the *latter* are not unhealthy, while at the same time the purgatives produce an increase of pain and tenesmus, with more copious discharges of blood and mucus, then they become, to say the least, unnecessary, perhaps detrimental, by increasing the irritability of the intestines, and determining a greater flow of blood to those parts. When purgatives are deemed necessary, our author has been in the habit of administering the neutral salts, with or without the infusion of senna.

*Emetics* our author has no experience of in dysentery, and does not approve of them.

*Sudorifics.* "Of all the general and constitutional remedies employed in the form of flux, now under consideration, this is the class of articles of which I have the most extensive experience, and to which I am disposed to assign the most powerful and salutary effects."

In this light he considers the employment of opium and ipecacuan, introduced into India by Mr. Abercrombie, of the 34th Regt. The practice was to give several grains of solid opium, following it by the exhibition of two or more ounces of infusion of ipecacuan. The other forms of sudorifics which our author chiefly employed were Dover's powder, and a combination of laudanum and antimonial wine; from all of which he observed beneficial effects. We would just here ask our author, if he conceives the form of dysentery now under consideration to be simply and purely inflammation of the colon, why opium and ipecacuan should not be equally beneficial in simple enteritis, of every-day occurrence? But here is the rock on which most writers on dysentery split. They find, when the disease proves mortal, inflammation and ulceration in the bowels, and they immediately conclude that the very last link in the chain of *effects* was the first in the chain of causes. In almost every case of fatal phrenitis, we find effusion of water at the base of the brain; but who, in his right senses, would set down hydrocephalus as the cause of phrenitis? So it is in dysentery. Inflammation and ulceration are secondary or ternary links in the morbid chain; and many a case of real dysentery is checked and cured, before either of these takes place—that is, when there is merely an increased afflux of blood to the mesenteric and portal vessels, a super-irritation in the mucous membrane of the bowels, and an increased discharge of acrid secretions from the intestinal glands. But to return.

*Warm Bath.* This remedy was found particularly useful in allaying pain, inducing sleep, diminishing the frequency of the stools, and promoting the discharge of urine. Fomentations to the abdomen, on the same principle, are serviceable.

*Mercury.* When the Regiment was first disembarked at Prince of Wales's Island, in full European health and vigour, mercury *alone,* carried to ptyalism, was not very successful; and truly we wonder, how men can be blind to the obvious inflammatory condition of the system at these times, and withhold the lancet, as auxiliary to the other means.

"I can readily conceive, and indeed know, that, in cases of a protracted disease, where the discharges from the intestines degenerate from pure blood and mucus, and become of a more diseased nature; that there is no remedy so much to be depended upon for the restoration of healthy secretions; but in the pure inflammatory complaint I am now speaking of, mercury can seldom be useful." P. 74.

*Topical Bleeding,* by leeches especially, is much recommended by Mr. B. on the authority of a letter from Dr. Annesley, Surgeon of the Madras European Regiment. Dr. Aitkin also suggested to our author the application of leeches to the anus in dysentery.

*Blisters* are spoken of as useful auxiliaries, whenever there is any fixed pain in any part of the colon.

*Injections.* These, of all remedies employed in dysentery, have appeared to our author, and to his patients, the most instrumental in alleviating the distressing tenesmus, diminishing the calls to stool, and lessening the profuse discharge of blood and mucus.—We can truly say that we have not found them so.

"In the composition of injections, decoctions of bark, solutions of the acetate of lead, and of the sulphate of zinc, were, at first, pretty extensively employed, and with a view of increasing their efficacy, were occasionally thrown up cold."

*Chronic or Hepatic Flux.* This our author looks upon, and justly too, as much more of a constitutional than a local disease. The circumstance of its prevailing among those who have been some time in the country; the degree of fever attending it; the diseased secretions in the stools, all evince functional derangement of the glandular viscera of the abdomen, as well as of the intestinal tube.

"That the functions of this organ [the liver] are, in most cases, materially, and perhaps primarily deranged, and that without a healthy action of this viscus, all our curative efforts will prove nugatory, are facts very generally, and as far as I know, most justly believed. P. 80.

*Mercury.* "If, in treating of the acute form of flux, I have refrained from an indiscriminate and, as I conceive, unmerited commendation of this powerful medicine, it is only in hopes of being able to urge its employment with double force in the form of disease now under consideration; to recommend an implicit reliance on it in the chronic form of flux; to ascribe to it an almost unlimited power in this disease; and to express an opinion, that it will seldom disappoint our most sanguine hopes. A partiality for the use of mercury is as conspicuous in India, as the aversion to blood-letting formerly noticed—that partiality is, however, much better founded." P. 81.

Almost every practitioner in India gives a preference to some particular preparation or form of the remedy; no weak proof, by the bye, how much depends on the medicine itself, and how little on the form of administration. If our author has formed a prepossession on this subject, it is in favour of the common blue pill. Before irritability of the stomach came on, Mr. B. thought that he could affect the system more speedily, and produce a change in the nature of the evacuations sooner, "by the daily exhibition of from twelve to twenty grains of the blue pill," than by any other preparation. Where gastric irritability prevails, or where mercury appears to affect the bowels, then he thinks mercurial frictions are preferable, as the system should be impregnated by rubbing in daily from one to two or three drachms of the blue ointment, according to the urgency of the symptoms, the rapidity with which the disease is proceeding, and the constitution of the patient.

"The exhibition of calomel, with opium, is a very favourite practice with many, and I have entered into this to a very considerable extent; three or four grains of calomel, and a grain of opium, made into a pill, and exhibited every three or four hours, I have soon found to produce all the beneficial effects resulting from the employment of mercury. P. 82.

The quantity will vary greatly in different individuals—

"But it is always to be carried the length of producing considerable ptyalism; and this, wherever the exhaustion of the patient does not forbid it, is to be kept up without intermission, until natural secretions return, and the stools resume a healthy appearance." P. 83.

But Mr. B. wisely avails himself of other auxiliaries in this form of dysentery. Purgatives, he thinks, are essentially necessary.

"The castor oil is a purgative in very extensive use amongst the natives of India, and many of the practitioners there give it a preference to every other."

This is the purgative, indeed, which we have always found to answer best in India. The warm bath and sudorifics are often useful to obviate the heat of the skin, and relieve the febrile symptoms in general, when they become urgent. Opiates, blisters, effervescing draughts, &c. are occasionally necessary; and tenesmus, he thinks, is best relieved by the anodyne glysters. The cummerband or belly band of flannel, is deservedly praised by our author.

"I have thus far endeavoured, both in the history and treatment, to show, that under the general denomination of dysentery or flux, we have two distinct forms of disease prevalent in India; and I now proceed to observe, that although, during the first years of my service in that country, these two diseases were often to be met with in practice as distinct as I have studied to keep them in description; they became latterly more and more blended together, and were to be found in all possible varieties of combination. This is what we should naturally expect from reasoning, and what is amply confirmed by my experience, so far as it goes. The well-known effects of warm climates, independent of the habitual use of spirits, will account for the existence of a liver affection in most of the cases of colonitis, which latterly recurred; while the well-known tendency of the country or pariar arrack (often rendered more deleterious by the admixture of acrid ingredients), to induce the acute or inflammatory flux, will account for the disposition latterly evinced by the hepatic fluxes, to terminate in inflammation of the colon. While the two forms of disease were thus frequently found co-existent, and running insensibly into each other, it was by no means uncommon to find them existing alternately for weeks or months, and destroying the patient by a form of flux, the symptoms of which alternately bore a nearer relation to one or other of the diseases I have described. These form a description of cases by far the most perplexing and troublesome we meet with; and, with respect to their treatment, the only general rule that can be laid down is, to urge the one or other mode of cure, in proportion as the one or other set of symptoms become more pressing.

And where the co-existence of both forms of flux renders it necessary to adopt a means of cure suited to this form of disease, we can only meet it by the simultaneous adoption of both modes of treatment. These, it will be observed, are by no means incompatible with each other; the one consisting chiefly in the exhibition of local remedies, directed to the lower part of the intestinal canal, while the other consists chiefly in the exhibition of mercury to affect the system. Had the appearance of one form of flux uniformly, or even generally, preceded the other, I should have been most ready to take the opportunity of considering them as different stages, rather than different forms of disease; but the want of uniformity in this respect leaves, in my opinion, no room for such a description." P. 89.

From the foregoing extract, our readers will be ready to suspect that the division of dysentery into colonitis and hepatic flux is rather fanciful than solid; and that the practical indications are full as well founded on the theory that dysentery is an increased irritation and afflux of blood to the mucous membrane of the intestines, with *functional* derangement in the liver, *ending* often in inflammation of the said membrane. On this account we have always considered the lancet as a material instrument in the treatment of dysentery; more, however, to prevent the *effects* than to remove the *causes* of this disease. Whoever encounters dysentery successfully, will aim at the restoration of the balance of the circulation and excitement, with the healthy *functions* of the skin and liver. He will do this, and guard at the same time against inflammation of the intestines by blood-letting, whenever pain on pressure of the abdomen, sanguineous discharge in the stools, and febrile movements in the system, indicate the necessity of this measure. The functions of the skin and liver will be best restored by calomel, opium, and ipecacuan, or antimony, assisted by the warm bath.

### LIVER COMPLAINTS.

When *acute* hepatitis occurs, it is neither difficult to recognize or treat it. It is infinitely less destructive than those insidious and ill-marked cases of chronic hepatitis which steal upon us unawares, and proceed to an extreme from which there is no chance of recovery, without giving either patient or surgeon the necessary degree of alarm.

" When we hear of patients dying suddenly from this disease, who were previously treated as *hypochondriacs*, and thought to enjoy a reasonable share of health; when we see abscesses in the liver discovered on dissection, which we never before dreamt of, it ought to awaken our most anxious inquiries, and impress upon our minds the necessity of an attention to the symptoms of those insidious attacks of liver complaint which reduce a patient to the brink of the grave, without giving him due warning of his danger." P. 91.

Although liver affections, in a great majority of instances, are the result of the conjoint effects of climate and intemperance; yet our author justly conceives, that they sometimes spring from climate alone. In the *latter* case, they are often very obscurely marked. Our author met but a few cases of the acute form of hepatitis in India, and they naturally led to an energetic or European practice; but his object, in this paper, is turned entirely to chronic hepatitis. Mr. B. confirms the observation made by preceding writers on tropical climates, that in the disease under consideration, the mind is clouded with an apprehension and alarm, which no external symptom seems to justify. The patient is generally overcome with a degree of languor, listlessness, and aversion to enterprize, without any adequate cause. Nor is this aversion to exertion caused by pain or inability; but he seems solely restrained by a state of mind rendering him indifferent to external objects, inducing him to paint the result of his complaints as inevitably fatal. He delights in detailing his miserable feelings to others, particularly to medical men; while it is obvious that he has the greatest difficulty in explaining his sensations accurately. Pain is not, in general, an urgent symptom; but when more distinctly complained of, is frequently described as occupying the epigastric, rather than the hypochondriac region. It is occasionally found extending up the right side, and reaching to the top of the right shoulder, where a gnawing or aching sensation is experienced. The patient generally speaks of a sense of oppression, fullness, or stuffing, about the lower part of the chest, which leads him to press upon the part, which gives a momentary relief. Sometimes, however, as was the author's case, a feeling of vacuity is experienced—a sensation as though a part of the liver were defective, or rendered entirely destitute of sensibility; and this disagreeable feeling is also relieved by pressure. A manual examination of the hepatic region will often assist us materially in ascertaining the existence of a liver affection, especially when emaciation has advanced to a certain point. Indeed, when the disease has been of considerable duration, an obvious swelling over the side will often be perceptible to the eye. When the patient is stripped, and minutely examined anteriorly and posteriorly, the ribs over the liver will frequently appear bulged out, as though by the act of inspiration. The pulse in chronic hepatitis does not afford any decisive criterion, until its increased quickness, accompanied by rigors, thirst, and restlessness, give reason to apprehend the formation of matter. A dry, tickling cough sometimes occurs; but it is not a symptom which generally attracts much of the patient's attention. An increase of pain, and difficulty of lying on the left side, when it exists, is a strong symptom of liver affection; but it is by no means constant—the back being often the easiest position. In almost every serious affection of the liver, there is a peculiar irritability of the stomach, marked by a degree of nausea and retching, sometimes vomiting of vitiated bile and phlegm, attended by a capricious, but not always defective appetite. After a full meal, a sense of tension and oppression in the region of the stomach is experienced, and the patient is sensible that he has taken too much food. This produces a degree of febrile thirst, restlessness, and intolerance of one position.

The state of the alvine secretions and excretions is, in every doubtful case of liver affection, to be most particularly attended to. They will be found, in most cases, materially altered, both as to quantity and quality. Our author has, under the head of dysentery, noticed one form of that disease, in which the liver is principally affected; and which might, perhaps with strict propriety, have been considered in this Section. Hepatitis, however, according to Mr. B. is not universally accompanied by a dysenteric state of the bowels. On the contrary, he thinks, the disease commences, in general, and sometimes exists for a certain time, with constipated bowels, the stools being sometimes of a darker, and sometimes of a lighter colour than natural; these colours frequently alternating for some length of time, and eventually terminating in flux. The stools often contain half digested substances, and a great extrication of flatus generally takes place.

"The urine, when examined, will be found depositing a copious reddish, flaky sediment; which, however, is a symptom not particularly confined to cases of hepatitis, but generally prevalent in all cases where the stomach is much affected, the digestion impaired, and the process of assimilation defective." P. 99.

The peculiar sallowness of complexion, and jaundiced eye, are so prevalent in India, among all classes, that, although perhaps originating in, or indicating a predisposition to liver affection, they cannot be taken much into consideration as diagnostic marks of disease.

In dissections of the acute form of hepatitis, it is very common to find one, or both lobes, almost wholly converted into matter, partly serous and partly purulent, confined in a sac formed by the peritoneal coat of the liver, which, in attempting to raise this organ from its position, is often ruptured, and the matter diffused over the abdomen. In some cases of this kind, the matter finds its way through the diaphragm into the lungs, and is discharged by expectoration; in many instances, an extensive adhesion will be found between the liver and transeverse arch of the colon, and through this adhesion the matter may, and often does burst into the intestinal canal, and is thence discharged by stool, as was once the case with the writer of this analysis.

In the chronic form of the disease, instead of meeting with an abscess of any remarkable size, we generally find several small, distinct collections of pus, similar to what are termed vomicæ in the lungs. The whole mass of the liver, in such cases, is generally altered in colour; while, in appearance, it looks as though it were parboiled, becoming much firmer in texture than natural; so much so, that on cutting into its substance, a sensation is communicated to the hand of the dissector, as though his knife were passing through a soft cartilaginous mass. The small quantity of blood which flows from an incision into an indurated liver of this description, is a remarkable feature, and affords a striking contrast to the state of liver existing in the remittent fevers of India. The appearance of white spots of various dimensions, and of indurated tubercles in its substance, is by no means uncommon in protracted and ill-defined cases of this disease. The diseased appearances, however, of the liver, as occurring in India, are generally confined to suppuration or induration, the legitimate results of preceding inflammation. The different species of tubera, as described by some recent authors, are by no means common occurrences in the hotter regions of the earth; and, from our own experience in post-mortem researches, they appear to be far from so common in this country, as they are represented. The changes in the bile itself, our author thinks, cannot be very considerable, though he has had no opportunity of analysing it. On this point, we do not agree with Mr. Ballingal, as we know, from attentive observation and personal feeling, that this fluid suffers the most marked and important deteriorations.

*Treatment.* Our author justly observes, that the primary object in the treatment of chronic hepatitis is to prevent suppuration, since recoveries, after such events, are rare indeed!

"From the deep seat of many of these abscesses, from the resistance opposed to their progress outwards, and from the ease [facility] with which the substance of the liver apparently gives way to the ulcerative process, we are often surprised to find an enormous collection of matter accumulated, before we have any external appearance to direct us to the precise seat of it, or to guide us in making an opening for its evacuation." 103.

In order to prevent this sinister accident, Mr. B. states, that he has employed blood-letting to a much larger extent than what the state of the pulse, the height of the symptoms, or the habit of the patient would seem to demand.

"Of this I can safely say, that I have never had occasion to repent; and that the more I practised it, the more confident I became in its use."

Mr. B. candidly confesses, however, that such is still the bigoted and formidable opposition with which the advocates of depletion have to contend in India, that it is almost impossible for the young practitioner to follow the dictates of reason or sound pathology.

The remedies next in efficacy are purgatives, of which "Calomel is generally and justly supposed to produce the most beneficial effects." "It is a common and effectual practice to administer six, eight, or ten grains of this preparation, in the form of pill, over night, and to follow it in the morning by the exhibition of a quantity of some of the neutral salts, the infusion of senna, or a dose of castor oil." P. 104.

In addition to this, if the purgatives appear tardy, their operation ought to be assisted by injections; and when, by these means, copious evacuations are procured, they seldom fail of producing the most beneficial effects, relieving the indescribable sense of tension, stuffing, and oppression, about the region of the liver, and making the patient feel comparatively light, easy, and cheerful.

We are not, however, to expect, in chronic hepatitis of any standing, that purgatives alone will succeed in restoring the structure and function of the organ to a sound state, and bringing the abdominal secretions to a healthy appearance.

"For these purposes, in every protracted case of this disease, the powers of mercury are alone to be trusted, and these are, in general, sufficiently efficacious. The relief, indeed, experienced in most cases of chronic hepatitis, the moment mercury affects the mouth, is truly surprising; the removal of all uneasy feelings from the side, the comparative clearness of the patient's skin and visage, the restoration of the natural evacuations, and the removal of every complaint but debility, evince the powers of this remedy; and were its effects always as permanent as they are conspicuous, we should have nothing farther to desire in the treatment of this complaint. Unfortunately, however, this is not one of those diseases to which a patient is only subjected once in his life. When, as is too often the case, the patient's circumstances compel him to remain in India, he is generally subject to a repetition of his complaints, and has often occasion to recur to the same remedy, which, like all others, loses its efficacy by frequent repetition." P. 106.

Our author prefers the blue pill in general, and wisely recommends leeches to the region of the liver in the incipient stages of hepatitis, with subsequent blisters, or a seton. Of the nitric acid, he can say little; and of the nitro-muriatic acid bath, nothing from personal experience. Upon the whole, the auxiliary remedies in chronic hepatitis are very imperfectly developed. There is not a single symptom or remedy mentioned in this paper of Mr. Ballingal's, that has not been urged by some late writers on tropical climates, and these Mr. B. ought to have perused. It is highly gratifying, however, to observe that our author, without recourse to books, has confirmed the leading features of some recent works on the same subject, and urged that mixed practice of depletion and mercury so necessary in diseases of the eastern hemisphere, but which have been stigmatised as incongruous, because not always necessary in the modified climate and constitution of the western world.

The extent to which we have carried our analysis, is the highest compliment we could pay our athor; and our estimation of the work may be calculated by the number of pages which we have dedicated to a faithful, and we trust an impartial delineation of its contents.

